# The Potential for Elimination of Racial-Ethnic Disparities in HIV Treatment Initiation in the Medicaid Population among 14 Southern States

**DOI:** 10.1371/journal.pone.0096148

**Published:** 2014-04-25

**Authors:** Shun Zhang, Shanell L. McGoy, Daniel Dawes, Mesfin Fransua, George Rust, David Satcher

**Affiliations:** 1 National Center for Primary Care, Morehouse School of Medicine, Atlanta, Georgia, United States of America; 2 Satcher Health Leadership Institute, Morehouse School of Medicine, Atlanta, Georgia, United States of America; 3 Office of the President, Morehouse School of Medicine, Atlanta, Georgia, United States of America; 4 Department of Medicine, Morehouse School of Medicine, Atlanta, Georgia, United States of America; Rollins School of Public Health, Emory University, United States of America

## Abstract

**Objectives:**

The purpose of this study was to explore the racial and ethnic disparities in initiation of antiretroviral treatment (ARV treatment or ART) among HIV-infected Medicaid enrollees 18–64 years of age in 14 southern states which have high prevalence of HIV/AIDS and high racial disparities in HIV treatment access and mortality.

**Methods:**

We used Medicaid claims data from 2005 to 2007 for a retrospective cohort study. We compared frequency variances of HIV treatment uptake among persons of different racial- ethnic groups using univariate and multivariate methods. The unadjusted odds ratio was estimated through multinomial logistic regression. The multinomial logistic regression model was repeated with adjustment for multiple covariates.

**Results:**

Of the 23,801 Medicaid enrollees who met criteria for initiation of ARV treatment, only one third (34.6%) received ART consistent with national guideline treatment protocols, and 21.5% received some ARV medication, but with sub-optimal treatment profiles. There was no significant difference in the proportion of people who received ARV treatment between black (35.8%) and non-Hispanic whites (35.7%), but Hispanic/Latino persons (26%) were significantly less likely to receive ARV treatment.

**Conclusions:**

Overall ARV treatment levels for all segments of the population are less than optimal. Among the Medicaid population there are no racial HIV treatment disparities between Black and White persons living with HIV, which suggests the potential relevance of Medicaid to currently uninsured populations, and the potential to achieve similar levels of equality within Medicaid for Hispanic/Latino enrollees and other segments of the Medicaid population.

## Introduction

There are nearly 50,000 new HIV infections each year and more than 1.1 million people living with HIV in the United States. Minority populations are disproportionately impacted by HIV/AIDS in the United States [1]. Black and Hispanic/Latino individuals comprise the majority of new HIV infections, and of people living with the disease in the United States [2]. Black or African American persons represented 44% and Hispanic/Latino individuals represented 20% of new HIV infections in 2009. Antiretroviral therapy has increased both the years and quality of life for people living with the condition.

The Centers for Disease Control and Prevention (CDC) released data in July of 2012 documenting the percentage of people in five stages on the HIV Care Continuum: diagnosed, linked to care, retained in care, prescribed ARV, and virally suppressed. Eighty-two percent (941,950) of the more than 1.1 million people living with HIV have been diagnosed. Sixty-six percent (725,302) have been linked to care. Thirty-seven percent (480,395) have been retained in care. Thirty-three percent (426,590) have been prescribed ARV. Twenty-five percent (328,475) have viral loads suppressed to less than or equal to 200 copies/mL [3], [4]. Minority (particularly Black and Hispanic/Latino) individuals, are diagnosed, linked to care, retained in care, prescribed ARV, and virally suppressed less than Whites [5].

There is considerable variation across states in HIV prevalence and treatment outcomes. The southern region of the United States has the highest burden of health disparities, particularly for HIV/AIDS [6]–[8]. In 2010, the southern region accounted for 37% of the population, yet 46% (21,592) of new HIV infection diagnoses [9]. Seven of the top ten states with the highest AIDS diagnosis rates and eight with the highest prevalence rates in 2010 were in the South (CDC). The percentages of early-stage diagnosis and survival for 36 months after a diagnosis of HIV infection between 2002–2006 were the lowest in the south [6]. Furthermore, Meditz et al. [10] found in a longitudinal study that more non-white and southern people living with HIV initiate antiretroviral therapy later and have greater HIV-related morbidity. Disparities in HIV/AIDS in the southern region of United States persist for a variety of reasons.

Advocacy organizations, grassroots mobilization efforts, HIV coalitions, and researchers have all sought to explain the disparate impact of HIV/AIDS in the South. Adimora et al. [11] articulate the social, structural, and policy dynamics that perpetuate HIV in the South by describing the rurality of the region, lack of providers with HIV specific treatment knowledge, distrust, stigma, policies surrounding such issues as Medicaid and sexual health education. Similar reasons for the disparities coupled with economic and behavioral health factors like poverty, homelessness, unemployment, substance abuse, and mental health challenges have been identified in the research literature, the Southern AIDS Coalition, and the Southern HIV AIDS Strategy Institute [6], [7]. To address areas greatly impacted by HIV/AIDS, the Centers for Disease Control and Prevention, Division of HIV/AIDS Prevention (DHAP) funded two demonstration projects, Enhanced Comprehensive HIV Prevention Planning (ECHPP) and Care and Prevention in the United States (CAPUS) [12]. CAPUS, a three year funded project, was designed to expand and improve HIV testing capacity, link, retain, and re-engage minorities is conducted in eight states, Georgia, Illinois, Louisiana, Mississippi, Missouri, North Carolina, Tennessee and Virginia [13]. Seven of the eight states with CAPUS demonstration projects are in our study. The goals of CAPUS align with the National HIV AIDS Strategy [14].

Federal efforts, the Ryan White HIV/AIDS Treatment and Extension Act of 2009, as well as state Medicaid programs provide some safety-net coverage for persons living with HIV, but financing for HIV/AIDS care has been insufficient to meet the growing need, and care coordination has been relatively fragmented [15]. Nevertheless, Medicaid is the largest source of coverage for persons living with HIV/AIDS. Established in 1965, Medicaid provides health coverage to more than 62 million low income Americans. In addition to income and asset eligibility limits, individuals must also meet categorical eligibility criteria, which include pregnant women, children, adults with dependent children, people with disabilities, and older adults (all of whom must also meet state-specific low-income criteria). Medicaid provides coverage not only for health professional services and health care facility costs, but all fifty states also include the optional benefit of prescription drug coverage [16].

Medicaid contributed $9.3 billion or 51% of all HIV care federal spending in 2011 [17]. An analysis of 2007 Medicaid Statistical Information System (MSIS) data from the Centers for Medicare and Medicaid Services (CMS) found Medicaid enrollees with HIV were less than 1% (212, 892) of all Medicaid enrollees, but they were nearly a quarter of all people diagnosed with HIV and nearly half of all people receiving regular HIV care. Moreover, Black and Latino individuals comprised nearly 70% of those with HIV enrolled in Medicaid for the full fiscal year of 2007 [17]. Consequently, Medicaid claims data provide a unique data source to investigate the initiation of ARV treatment among minority populations, and constitute a potential surveillance system for tracking progress toward the elimination of disparities in treatment and outcomes. The purpose of this study is to explore the racial/ethnic disparities in the initiation of antiretroviral treatment among HIV-infected Medicaid enrollees in 14 southern states.

## Methods

### Study Design

A retrospective cohort design was used to explore racial/ethnic disparities in the initiation of ARV treatment among HIV-infected Medicaid enrollees. Recommendations from the 2007 U.S. Department of Health and Human Services Panel on Antiretroviral Guidelines for Adults and Adolescents, regarding opportunistic infections and pregnancy, with the exclusion of CD4 T-cell counts and viral loads, were used to identify persons who should begin ARV treatment [18].

Data for this analysis were taken from the Medicaid claims data from fourteen southern states between 2005 and 2007. All data were deidentified to protect the privacy of individual patients, physicians, and hospitals.These data are restricted to use for an approved project by designated researchers for a limited time period under a confidential data use agreement with the Centers for Medicare & Medicaid services. Other researchers may apply to the CMS privacy board for a similar data use agreement. The study was approved for human subjects research by the medical school’s Institutional Review Board.

### Data Source

We used MAX data from fourteen southern states (Alabama, Arkansas, Florida, Georgia, Kentucky, Louisiana, Maryland, Missouri, Mississippi, North Carolina, South Carolina, Tennessee, Texas, and Virginia) representing paid claims for encounters occurring during calendar years 2005 through 2007. We included non-elderly adults age 18–64 with a diagnosis of HIV/AIDS. Our analyses used four of the five available Medicaid Analytic Extract (MAX) files for each state and each year: 1) Personal Summary File; (2) Inpatient File; (3) Out-patient File; and (4) Prescription drug file. We also combined Medicaid claims data with county level contextual data on socioeconomic and environmental characteristics from the Area Resource File (ARF). Federal Information Processing Standard (FIPS) codes for patient’s county of residence were used to merge the ARF and MAX files.

### Participant selection

The International Classification of Disease, ninth Revision, Clinical Modification (ICD-9-CM) code for HIV/AIDS (042.XX, V08.XX, 795.71) was used to identify HIV positive persons in the dataset. A total of 102,782 Medicaid enrollees were found to have at least two outpatient claims or one inpatient claim with a diagnosis of HIV/AIDS in the three year Medicaid claims dataset. A limitation in the participant selection is that CD4 and viral load data are not available in Medicaid claims data; for this reason, we used the U.S. DHHS Guidelines for the Use of Antiretroviral Agents in HIV-1-Infected Adults and Adolescents 2007 to identify those individuals who should begin ARV treatment [18]. Criteria used to identify HIV/AIDS positive persons with specific clinical indications for ARV treatment included those with opportunistic infections, pregnant women, individuals with HIV-Associated Nephropathy (HIVAN), and Hepatitis B virus (HBV) co-infected persons. We identified those conditions based on diagnosis codes (related ICD-9 codes listed in supplement) on paid claims in the Medicaid inpatient and out-patient files.

**Figure 1:**
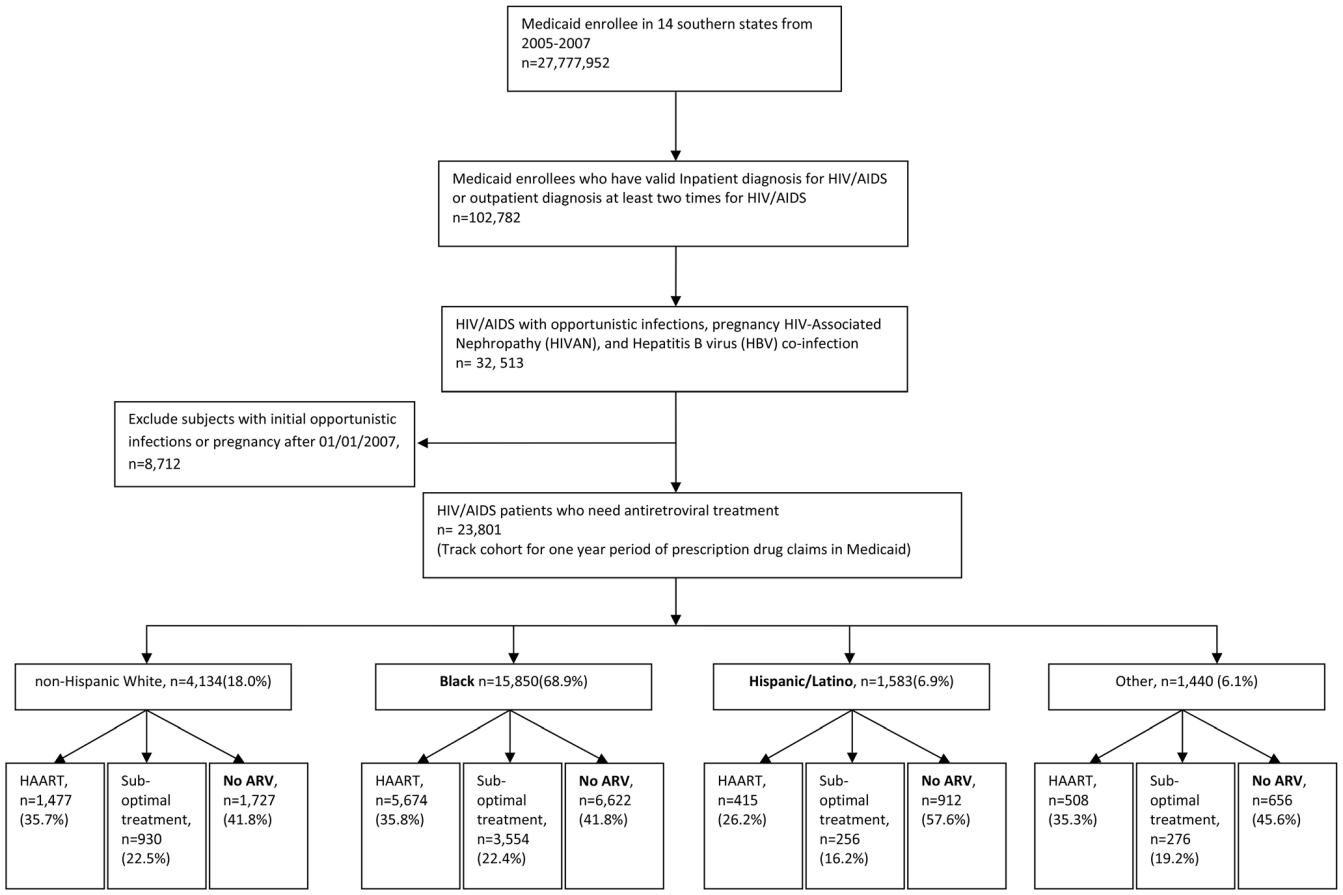


There were 32,513 HIV-positive participants with specific indications for ARV-therapy according to 2007 treatment guidelines in the final study cohort. In order to allow for a one-year tracking period of ARV treatment usage, individuals were excluded who had an initial HIV diagnosis after January 1st 2007. The final sample size was 23,801. (Figure 1).

### ARV Treatment definition

We used the National Drug Code (NDC) variable in prescription drug claims to identify specific ARV treatments. Five types of FDA approved ARV drugs were currently recommended and available in 2007: (1) nucleoside reverse transcriptase inhibitors (NRTIs) including zidovudine, didanosine, stavudine, lamivudine, abacavir, emtricitabine,and tenofovir; (2) non-nucleoside reverse transcriptase inhibitor (NNRTIs) including efavirenz, nevirapine, delavirdine, and etravirine; (3) protease inhibitor (PIs) including atazainavir, darunavir, fosamprenavir, indinavir, nelfinavir, ritonavir, saquinavir, tipranavir, and lopinavir; (4) entry inhibitors including enfuvirtide and maraviroc; and (5) one integrase inhibitor (raltegravir). An optimal ARV regimen accepted as Highly Active Antiretroviral Therapy (HAART) or optimal ART during the study period was defined as either one NNRTI or a PIs and two NRTIs. A “sub optimal treatment” group was defined by the use of any other patterns of ARV treatment except for the optimal treatment. Patients who had no claims for ARV drugs during the one year period after demonstrating treatment indications were defined as the “no-ARV” treatment group.

### Independent variables

Rural/Urban Status was determined by merging the MAX data with county level data from the Area Resource File (ARF) [19]. The ARF aggregates publically available data from multiple sources about socioeconomic and environmental characteristics. Federal Information Processing Standard (FIPS) codes for patient’s county of residence were used to merge the ARF and MAX files. The 2003 Rural/Urban Continuum Codes are from the Department of Agriculture’s Economic Research Service (ERS) [20], classifying counties into three groups: Large metro area with at least 1 million residents or more; Small metro area with fewer than 1 million residents; and Non-metro (rural) areas.

Medicaid enrollment months were averaged from 3 years of aggregated Medicaid claims data into a per year scale. We then classified enrollment months into groups based on the number of months of Medicaid enrollment per year (1–3 months per year, 3–6 months per year, 6–9 months per year, and 9–12 months per year).

### Analytic Procedures

Descriptive analyses of variance of numerical variables by race/ethnicity were measured by ANOVA. Frequency variances among different racial-ethnic groups and different treatment groups were compared by using the Chi-square (χ^2^) test. Multinomial logistic regression models used HAART (optimal ART) as the comparison group. The univariate and multivariate model was used to estimate the relationship between covariates and different treatment group. The unadjusted odds ratio for accessing No ART vs. HAART and Sub-Optimal Treatment vs. HAART was estimated through multinomial logistic regression using race/ethnicity and other variables as a single independent variable. The multinomial logistic regression model was repeated with adjustment for multiple covariates, which included age, state, rural/urban status, and Medicaid enrollment months status. Using non-Hispanic White as the reference group, a 95% confidence interval was estimated for African-Americans, Hispanics, and "Other" racial group. The level of statistical significance was set at 0.05 and all tests were two tailed. Analyses were conducted using SAS 9.2 (Cary, NC).

## Results

Table 1 describes the characteristics of the study population. Of the total sample of 23,801 Medicaid HIV-infected enrollees who meet the established criteria to begin ARV treatment Blacks and Hispanics/Latinos comprised nearly 75% of the individuals in the study. More than half (12,649, 53.1%) of the study population lived in large metro areas. The average of Medicaid eligible months per year for Hispanic/Latino persons was lowest at 4.5 months compared with 7.2 months per year for White persons, 7.6 months per year for Black persons. Moreover, almost half (48.6%) of Hispanic/Latino persons were only Medicaid-eligible for 1–3 months per year.

**Table 1:** Characteristics and Treatment of 23,801 HIV-infected Medicaid enrollees, 14 southern states, 2005–2007.

	White,N (%)	Black,N (%)	Hispanic/Latino, N (%)	Other,N (%)	P-value
N(%)	4134(18.0%)	15850(68.9%)	1583(6.9%)	1440(6.2%)	***
Age					
mean, (sd)	40.7(9.9)	39.8(11.2)	38.1(11.6)	40.8(12.9)	<.01
Age group					
< = 18 yrs	54(1.3%)	402(2.5%)	42(2.7%)	239(16.6%)	<.01
18–45	2836(68.6%)	10572(66.7%)	1155(73.0%)	714(49.6%)	
>45	1244(30.1%)	4876(30.8%)	386(24.4%)	487(33.8%)	
Metro index					
Large Metro	2120(53.0%)	9085(60.9%)	784(63.5%)	660(56.2%)	<.01
Small Metro	1251(31.3%)	4148(27.8%)	371(30.0%)	356(30.3%)	
Non Metro	630(15.8%)	1677(11.3%)	80(6.5%)	159(13.5%)	
Eligible months per year					
mean,(sd)	7.2(3.9)	7.6(3.9)	4.5(3.9)	6.3(4.3)	<.01
months eligible per year groups					
1–3 months	891(21.6%)	2984(18.8%)	770(48.6%)	466(32.4%)	<.01
3–6 months	772(18.7%)	2799(17.7%)	218(13.8%)	225(15.6%)	
6–9 months	1034(25.0%)	3661(23.1%)	396(25.0%)	312(21.7%)	
9–12 months	1437(34.8%)	6406(40.4%)	199(12.6%)	437(30.4%)	
Treatment					
HAART *	1477(35.7%)	5674 (35.8%)	415 (26.2%)	508 (35.3%)	<.01
Sub Optimal Treatment**	930(22.5%)	3554(22.4%)	256(16.2%)	276(19.2%)	
No ART	1727(41.8%)	6622 (41.8%)	912 (57.6%)	656 (45.6%)	

*HAART Treatment: either one NNRTI (non-nucleoside reverse transcriptase inhibitor) or a PI (protease inhibitor) and two NRTIS (nucleoside reverse transcriptase inhibitors).

**Sub-Optimal Treatment: Some or any ARV treatment prescription rather than HAART.

***794 Medicaid enrollees missed the race ethnicity classification in the dataset.

Table 2 shows descriptors of those receiving HAART, sub-optimal treatment, and No ART. Nearly forty-four percent (10,449) of the study population did not receive any ARV treatment during the one year period after meeting established criteria or indications for ARV treatment initiation. Black and White sub-populations had nearly identical proportions of individuals who received different treatment. There were less Hispanic/Latino persons who received HAART and sub optimal treatment, and more than half Hispanic/Latino did not receive any ARV. More than two thirds (68%) of persons who were enrolled in Medicaid only 1–3 months per year did not receive any ARV treatment, compared to less than one third (31.9%) of persons who had 9–12 months per year Medicaid enrollment did not receive ARV treatment.

**Table 2:** Antiretroviral Drug Treatment of HIV infected Medicaid enrollees for 2005–2007, 14 southern states.

	total	HAART	Sub-optimal treatment	No ART	P-value
Total	23801	8228(34.6%)	5124(21.53%)	10449(43.9%)	
Race/ethnicities					
White, NH	4134	1477(35.7%)	930(22.5%)	1727(41.8%)	<.01
Black, NH	15850	5674(35.8%)	3554(22.4%)	6622(41.8%)	
Hispanic/Latino	1583	415(26.2%)	256(16.2%)	912(57.6%)	
Other	1440	508(35.3%)	276(19.2%)	656(45.6%)	
Age Group					
< = 18 yrs	1531	543(35.5%)	227(14.8%)	761(49.7%)	<.01
18–45 yrs	15277	5089(33.3%)	3649(23.9%)	6539(42.8%)	
>45 yrs	6993	2596(37.2%)	1248(17.9%)	3149(45.0%)	
Urban/Rural					
Large Metro	12649	4312(34.1%)	2729(21.6%)	5608(44.3%)	<.01
Small Metro	6126	2137(34.9%)	1426(23.3%)	2563(41.8%)	
Rural	2546	925(36.3%)	563(22.1%)	1058(41.6%)	
Month enrolled per year in Medicaid					
1–3 months	5905	959(16.2%)	911(15.4%)	4035(68.3%)	<.01
3–6 months	4014	1207(30.1%)	1060(26.4%)	1747(43.5%)	
6–9 months	5403	2168(40.1%)	1271(23.5%)	1964(36.4%)	
9–12 months	8479	3894(45.9%)	1882(22.2%)	2703(31.9%)	

Binary logistic regression models (No ART vs. HAART, and Sub-optimal Treatment vs. HAART) were used for the analysis in Table 3. Hispanic/Latino persons had nearly 2 times greater risk (1.88 Crude RR, 1.64, 2.15) of not receiving ARV vs. HAART compared with Whites. HIV positive persons who had 1–3 months per year Medicaid eligibility had more than 6 times greater risk (5.56, 6.61) of not receiving ARV vs. HAART compared with those who had 9–12 months per year Medicaid eligibility. The same pattern was seen in the Sub-optimal treatment vs. HAART. After adjusting for covariates, age, state, Hispanic ethnicity, Medicaid eligible months per year remained as factors that influenced access to ARV treatment the year after the patient met clinical indications for ARV treatment. While there were no observed Black-White racial disparities in treatment, Hispanic/Latino persons were 45% (1.22, 1.73) more likely to not to receive ARV vs. HAART than Whites non-Hispanic persons.

**Table 3:** Crude and Adjusted Relative Risks and 95% Confidence Interval from Multinomial Logistic Regression for the Relationship between Covariates and Different HIV Medicaid enrollees Treatment Groups, 14 southern states, 2005–2007.

	No ART vs. HAART				Sub-optimal Treatment vs. HAART			
	Crude		Adjusted		Crude		Adjusted	
	RR	95% CI	RR	95% CI	RR	95% CI	RR	95% CI
Race(ref = white)								
Black	1.00	(0.92,1.08)	1.05	(0.96,1.15)	1.00	(0.91,1.09)	1.04	(0.94,1.15)
Hispanic	1.88	(1.64,2.15)*	1.45	(1.22,1.73)*	0.98	(0.82,1.17)	0.89	(0.72,1.10)
Other	1.10	(0.97,1.26)	0.97	(0.83,1.13)	0.86	(0.73,1.02)	0.88	(0.73,1.06)
Age Group (ref = <18 yrs)								
18–45 yrs	0.92	(0.82,1.03)	2.47	(1.99,3.07)*	1.71	(1.46,2.01)*	2.63	(2.05,3.37)*
>45 yrs	0.87	(0.77,0.98)*	2.57	(2.06,3.20)*	1.15	(0.97,1.36)	1.82	(1.40,2.35)*
Metro index(ref = Large metro)								
Small metro	0.92	(0.86,0.99)*	0.91	(0.83,0.99)*	0.91	(0.83,0.99)*	1.01	(0.91,1.11)
Non metro	0.88	(0.80,0.97)*	0.88	(0.79,0.99)*	0.96	(0.86,1.08)	0.93	(0.82,1.06)
Medicaid enrollment months per year(ref = >9–12 months per year)								
1–3 months	6.06	(5.56,6.61)*	6.33	(5.73,7.00)*	1.97	(1.77,2.19)*	2.09	(1.85,2.36)*
3–6 months	2.09	(1.91,2.28)*	2.17	(1.98,2.39)*	1.82	(1.65,2.01)*	1.78	(1.60,1.98)*
6–9 months	1.31	(1.21,1.41)*	1.58	(1.45,1.73)*	1.21	(1.11,1.33)*	1.35	(1.22,1.49)*

*significant difference (P<.05).

## Discussion

The findings from this study demonstrate the elimination of black-white racial disparities in HIV-treatment initiation in the Medicaid population of fourteen high-disparity southern states, but a persistent ethnic disparity in the initiation of ARV treatment among Hispanic and Latino HIV-infected Medicaid enrollees. Overall, HAART treatment levels for all segments of the population were less than optimal.

Reducing HIV/AIDS-related disparities and health inequities is one of three aims of The White House’s National HIV/AIDS Strategy (NHAS) released in July 2010. The strategy proposes to reduce HIV/AIDS-related mortality in high risk communities and among gay and bisexual men, as well as among Blacks and Latino persons, by 2015. Progress toward achieving mortality reductions will be measured by increases in undetectable viral loads by 20 percent in these populations [14], an objective which can only be achieved by increasing effective use of optimal ARV treatment. Hall et al reported that in the overall U.S. population in 2009, 35% of white but only 29% of black persons with HIV had ever been prescribed ARV (treatment initiated) [21]. The fact that black-white racial disparities in treatment initiation have been eliminated in the Medicaid populations of fourteen southern states is a profound affirmation of the possibility of achieving equality in treatment and outcomes. In this case, Medicaid matters!

This is especially relevant as the Patient Protection and Affordable Care Act 2010 (ACA) goes into effect, with over 16 million uninsured individuals expected to gain health insurance through Medicaid expansion in 2014. This is intended to increase access to care for individuals up to 65 years of age with incomes up to 138% of the federal poverty level (FPL), eliminating the categorical eligibility requirements such as pregnancy or disability. However, the expansion is contingent upon each state’s decision to expand Medicaid coverage. Inconsistencies in state expansion of Medicaid could represent missed opportunity to achieve greater equality in HIV/AIDS care. Our data demonstrate that the expansion of Medicaid has the potential to eliminate racial disparities in the initiation of antiretroviral treatment, and could also represent a public health surveillance tool for monitoring not only initiation.

HIV/AIDS is disproportionately affecting the Hispanic/Latino population as well. In 2009, Latino persons comprised 20% of the new HIV/AIDS cases in the United States [1]. The disparities in ARV treatment among Hispanic/Latino persons demonstrated in this study findings suggest the need to increase undetectable viral loads among this group, but there are cultural and social barriers that make increases in undetectable viral loads among Hispanic/Latino persons challenging. Key elements are to decrease structural barriers to continuous and prolonged Medicaid enrollment, as well as to assure that equal treatment is provided regardless of cultural, linguistic, or other patient characteristics. In our study, Hispanic or Latino individuals had exactly the same Medicaid card, scope of benefits, payment rates, low-income eligibility rules, provider networks, and drug formularies as non-Hispanic individuals, but did not receive appropriate treatment at the same rates. More than half did not receive ARV treatment (57.6%), and were nearly two times (AOR 1.88, [1.64, 2.15]) less likely to receive ARV treatment. Quality of care can be negatively impacted by language barriers [22]. The lack of bilingual providers can hinder the treatment and care experience of non-English speaking Hispanic/Latino patients. Socioeconomic factors like income, educational attainment, and lack of transportation can also be barriers to effective treatment [23]. Mistrust and cultural disconnects between the patient and provider interaction can also delay treatment. Stone identified differences in provider behaviors (e.g., prescribing medications to minority patients), based on provider views that minority patient adherence to HAART may be lower [24].

One specific structural barrier experienced by Hispanic and Latino patients was that nearly 50% were enrolled in Medicaid only 1–3 months during a given calendar year. This can be tied to immigration policies that hinder access to treatment and care, including The Personal Responsibility and Work Opportunity Reconciliation Act (PRWORA) of 1996 [25]. It requires persons who gain permanent residence in the United States to wait a period of 5 years to receive such services as Medicaid [26]. This policy disadvantages many HIV infected persons, particularly women of child bearing age. The chance that HIV infection will be transmitted from an HIV-infected pregnant woman to her child can be reduced to two percent or less with treatment [27]. Zhang et al. [28] found that among HIV-infected pregnant Medicaid enrollees in fourteen states in 2005–2007, 74.0% of the Hispanic/Latina women received no prenatal antiretroviral treatment. It is likely that the incidence of HIV/AIDS can be significantly reduced from mother to child if the mothers received comprehensive prenatal care, including HIV testing, and ARV treatment. Extending antiretroviral treatment to all HIV-infected pregnant persons in the U.S. regardless of citizenship status could also reduce the chances of vertically-transmitted HIV infection for such children, who will be born as an U.S/citizens [29]. In 2010, changes to the Illegal Immigration Reform and Immigrant Responsibility Act (IIRIRA) of 1996 made individuals with communicable diseases such as HIV/AIDS no longer a criterion for inadmissibility and deportation [26], [30]. The Deferred Action for Childhood Arrivals (DACA) provides deferred removal or deportation from the U.S. to individuals who meet specific criteria, mostly those who arrived in the U.S. before 16 years of age and those 31 years of age and under as of June 15, 2012 [31]. However, the Patient Protection and Affordable Care Act of 2010 (ACA) prohibits undocumented persons from being eligible for public-funded health insurance benefits, and specific regulations prohibit DACA beneficiaries from participating in ACA related programs or Medicaid [32]. These policies greatly impact antiretroviral treatment among Hispanic/Latino individuals.

There are several important limitations inherent in this study. Medicaid claims data are generated for administrative and reimbursement purposes rather than for clinical care or health services research. Medicaid claims data do not include individual covariates such as viral load, duration and severity of illness, socioeconomic status, education level, country of origin, length of stay in the US, or degree of social support, all of which may contribute to ARV access and health care utilization. Duration of Medicaid enrollment did not completely account for racial and ethnic differences. Even so, Medicaid enrollees of all racial/ethnic groups must meet similar low income criteria to enroll in the program within a given state. Important clinical variables such as CD4+ count and viral loads could not be controlled for in the analyses. Additionally, HIV and Hepatitis B Virus (HBV) co-infected patients were 8.8% (2805/23801) of the cohort; however, not all HBV infected persons were eligible for HAART. The 2007 treatment guidelines advised that HIV and HBV co-infected persons who had indications for HBV treatment should start HAART to treat both infections, instead of antiviral medications targeting only HBV. NRTIS such as Tenofovir (TDF), Lamivudine (3TC) and Emtricitabine can treat both HIV and HBV, while an antiviral such as Entecavir is approved for HBV treatment only. Entecavir can induce M184V mutation in the HIV genome, enabling the virus to resist Lamivudine and Emtricitabine when used in HIV and HBV co-infected patients as a mono-therapy. The treatment guidelines were designed to discourage Entecavir as mono-therapy in HIV and HBV co-infected patients. Finally, the Medicaid claims data in this analysis only encompassed 14 southern U.S. states, selected based on their large minority populations and disproportionate contribution to U.S. racial and ethnic disparities.

Notwithstanding these limitations, this study is one of the first to analyze racial and ethnic disparities in ARV treatment of initiation for HIV-infected patients using a multi-state Medicaid population. Our data show that within the Medicaid population racial equality in treatment initiation is achievable, but the data also identify Hispanic/Latino patients as a specific sub-group at risk of inadequate ARV treatment, in part related to the systematic exclusion of many immigrants from Medicaid-covered care. Finally, our data suggest the potential for Medicaid claims data to provide an on-going surveillance system of ARV treatment of HIV in high-disparity segments of the population.

## Conclusions

The impact of Medicaid on the elimination of HIV treatment disparities between Black and White persons suggests that treatment disparities are not inevitable, and that equality is achievable for Hispanic/Latino and other populations if policies are focused on achieving equitable population health outcomes for all. Medicaid expansion to all low-income persons regardless of disability or immigration status could substantially increase access to optimal and equitable care for all persons living with HIV.

## Supporting Information

Table S1 ICD9-9CM CODES FOR AIDS INDICATOR DISEASES.
(DOCX)
