# Supplementary material for: The Potential for Elimination of Racial-Ethnic Disparities in HIV Treatment Initiation in the Medicaid Population among 14 Southern States
Source: PLoS One. 2014 Apr 25;9(4):e96148. doi: 10.1371/journal.pone.0096148 (PMC4000218; doi:10.1371/journal.pone.0096148)
Supplement: Table S1 — ICD9-9CM CODES FOR AIDS INDICATOR DISEASES. (DOCX) [file pone.0096148.s001.docx]

**ICD9-9CM CODES FOR AIDS INDICATOR DISEASES**

|  | Bacterial infections – infection bacteria | 041.89 |
| --- | --- | --- |
|  | Bacterial infection unspecified | 041.9 |
|  | Candidiasis, bronchi, trachea, or lungs | 112.4 (lungs)  112.89 (other) |
|  | Candidiasis, esophageal | 112.84 |
|  | Carcinoma, invasive cervical | 180.9 (cervical)  195.0 (cervical region) |
|  | Coccidioidomycosis, disseminated or extrapulmonary | 114.1 (extrapulmonary)  114.3 (disseminated) |
|  | Cryptococcosis, extrapulmonary | 117.5 |
|  | Cryptosporidiosis, chronic intestinal (>1 month duration) | 007.4 |
|  | Cytomegalovirus disease (other than in liver, spleen or nodes) | 078.5 |
|  | Cytomegalovirus retinitis (with loss of vision) | 363.20 |
|  | HIV encephalopathy | 348.3 |
|  | Herpes simplex; chronic ulcer(s) (>1 month duration); or bronchitis, pneumonitis or esophagitis | 054.9 and 054.79 |
|  | Histoplasmosis, disseminated or extrapulmonary | 115.99 |
|  | Isosporiasis, chronic intestinal (>1 month duration) | 007.2 |
|  | Kaposi’s sarcoma | 176.9 |
|  | Lymphoma, Burkitt’s (or equivalent term) | 200.20 |
|  | Lymphoma, immunoblastic (or equivalent term) | 200.80 |
|  | Lymphoma, primary in brain | 202.80 |
|  | Mycobacterium avium complex or M. kansasii, disseminated or extrapulmonary | 031.2 |
|  | M. tuberculosis, pulmonary | 011.9 |
|  | M. tuberculosis, disseminated or extrapulmonary | 018.9 |
|  | Mycobacterium, of other species or unidentified species, disseminated or extrapulmonary | 031.2 |
|  | Pneumoncystis carinii pneumonia | 136.3 |
|  | Pneumonia, recurrent, in 12 month period | 486 |
|  | Progressive multifocal leukoencephalopathy | 046.3 |
|  | Salmonella septicemia recurrent | 003.1 |
|  | Toxoplasmosis of brain | 130.0 |
|  | Wasting syndrome due to HIV | 799.4 |
|  | AIDS | 042 |
|  | Non-specific serologic evidence of HIV | 795.71 |
|  | Asymptomatic HIV Infection Status | V08 |
|  |  |  |
|  | HIV-Associated Nephropathy (HIV AN) | 042A |
|  | Hepatitis B virus (HBV) co-infected persons | 070.3 |
|  | Pregnant Women |  |
